# A Treatise on the Diseases and Surgical Operations of the Mouth and Parts Adjacent: With Notes of Interesting Cases, Ancient and Modern

**Published:** 1851-04

**Authors:** 


*A Treatise on the Diseases and Surgical Operations of the Mouth and Parts Adjacent: with Notes of Interesting Cases, Ancient and Modern.* By M. Jourdain, Dentist, and Member of the College of Surgery. Translated from the last French edition. Philadelphia: Lindsay & Blakiston, 1851.

For this addition to American medical literature, we are indebted to Dr. Philip H. Austen, of this city, whose unreasonable modesty has prevented the appearance of his name upon the title page. If the writer of a good book lays his readers under an enduring obligation, the translator, who takes it from the literature of one people and incorporates it into that of another, making it available to multitudes who, without his aid, would have been nothing the better for its existence, is certainly worthy of commendation and gratitude. The measure of approbation must be found in the value of the literary gift, and that is made up of the importance of the original and the excellence of the translation. Judged thus, Dr. Austen has bestowed upon American surgeons, and particularly upon surgeon dentists, a most useful gift. The original work is exceedingly valuable, and his translation of it remarkably well done. The Baltimore College of Dental Surgery may be proud of a pupil who has commenced his career by such a contribution to the literature of the profession.

The translator began the work with the intention of adapting it, by suitable additions and corrections, to the present wants of the dental profession, and this design he carried out through the first hundred pages; but finding that the necessary additions would swell the book beyond a prudent size, he abandoned this part of the plan.

This is much to be regretted, for the additions thus partially made, are of such a character as to warrant the assurance that the whole work would have been greatly improved by persevering in the plan originally contemplated.

Of Jourdain's work it would be unnecessary to express an opinion, had not its foreign vesture hitherto hidden it from most of those who have sought information in this department of medical science. It is an old work—having been written about seventy years ago—yet it has never been, and, in many respects, never can be, superseded by another. In view of the low state of dentistry at the time of Jourdain, the production of such a book as his is wonderful; yet it is more wonderful that its publication did not lead at once to a higher appreciation of the dental art, and more diligent devotion to its improvement.

Jourdain at first studied general surgery with great diligence, but becoming embarrassed with the multitude of subjects embraced in manual medicine, he resolved to confine his business to the treatment of maladies of the mouth and adjacent parts. In order to acquire skill in this specialty, he read carefully all that had been written upon the subject; obtained all the information he could from living surgeons, and added to all the knowledge thus procured, the results of long and patient and widely spread observation.

This work contains, within a brief space, the aggregate of all his knowledge upon these subjects; and a wonderful aggregate it is. Making due allowances for the improvements in pathology and therapeutics made since the time in which he wrote, Jourdain's work is as useful now as it ever was, and every surgeon in the country would be well repaid for reading it. To the dental surgeon it is particularly valuable, and to such we earnestly recommend it. Dentistry, as yet, can boast of few books, but they are, for the most part good: let them be well studied, and none more closely and generally than Jourdain's.

